# The effect of the COVID-19 pandemic on the baby-friendly community initiative and maternal infant and young child nutrition in Kenya

**DOI:** 10.1186/s12889-025-22670-y

**Published:** 2025-05-01

**Authors:** Antonina Namaemba Mutoro, Milka Wanjohi, Calistus Wilunda, Maureen J Koech, Ajibola Ogunsola, Antuela Tako, Thomas Gyuchan Jun, Patrick Waterson, Paula L Griffiths, Elizabeth Wambui Kimani-Murage

**Affiliations:** 1 African Population and Health Research Center, Aphrc Campus, Manga Cl, Nairobi, Kenya; 2 Loughborough University, Loughborough, UK

**Keywords:** COVID-19, Maternal infant and young child nutrition, Baby-friendly community initiative, Maternal and child health

## Abstract

**Background:**

The COVID-19 pandemic led to decline in access and utilization of the baby-friendly community initiative (BFCI) which is being implemented in Kenya. The impact of the pandemic on the BFCI and on maternal and child health and nutrition has not been documented. We undertook a qualitative study that assessed the effect of the COVID-19 pandemic on the baby-friendly community initiative (BFCI) activities, maternal and child health (MCH) services and maternal, infant and young child nutrition (MIYCN) practices in Kenya.

**Methods:**

Data on the impact of the pandemic on the BFCI activities, provision and access to MCH services and MIYCN practices were collected using key informant interviews (*n* = 57), in-depth interviews (*n* = 31), and focus group discussions (*n* = 15) with government officials, civil society organizations and community members in BFCI implementing and non-implementing urban and rural areas.

**Results:**

Our study found that BFCI activities, such as home visits, support group meetings and MCH services such as nutrition counselling, growth monitoring and vaccination were interrupted by the pandemic due to fear of contracting the virus, lack of personal protective equipment (PPEs) and movement restrictions. This meant that mothers did not have access to basic community and health services. Food insecurity attributed to financial difficulties resulted in coping strategies such as skipping meals and negatively affected MIYCN practices. Positive measures to prevent COVID-19 spread such as remote working enabled some mothers to adequately feed their children because they were better able to balance working and the demands of feeding young children from home.

**Conclusion:**

On balance, the pandemic negatively impacted the BFCI, MCH services and MIYCN practices in Kenya. In such a context, there is a need for innovative approaches to ensure continued provision of and access to facilities and community health services in the future if the country finds itself in a similar position with the challenges of a pandemic. The pandemic revealed that remote working support policies could have the potential to improve breastfeeding and complementary feeding for working women but further evidence is needed to fully evaluate this.

## Introduction

The COVID-19 pandemic was expected to have a negative impact on maternal infant and young child nutrition (MIYCN) outcomes due to the preventive public health measures put in place by most governments including Kenya, which limited access to and utilization of essential MIYCN services such as breastfeeding and nutrition counselling, micronutrient supplementation and treatment of malnutrition [1–3] A UNICEF report indicated that low- and middle- income countries (LMICs) experienced a decline in nutrition services, which is likely to jeopardize vital nutrition gains that have been made over the past years [1]. In Kenya for example there were reports of declined attendance to MCH clinics, lack of clear guidance on breastfeeding during the pandemic and limited one on one counselling of mothers due to the COVID-19 restirctions [2, 3]. In Bangladesh and India significant declines in service provision, especially services which require patients to be near health facilities and health personnel such as counseling on child feeding and food supplementation, have been reported because of the COVID-19-induced lockdowns [4]. Health care providers in Kenya and other African countries limited access to child health services such as growth monitoring during the COVID-19 period [5, 6]. Lockdowns negatively affected the running of many MIYCN interventions and programs, forcing these programs to scale down operations, thereby leading to reduced service provision and utilization [7, 8]. For example, in Brazil and the UK, support for kangaroo mother care was reduced and early initiation of breastfeeding was discouraged at the onset of the pandemic due to the strict prevention measures that were enforced [9, 10]. Such practices are likely to have reduced exclusive breastfeeding rates.

Significant improvements in maternal, infant, and young child feeding practices were reported globally before the pandemic. In Kenya for example, results from the 2014 Kenya Demographic and Health Survey show that 62% of infants were breastfed within the first hour of delivery, 91% were breastfed within a day of birth and about two-thirds (61%) of children under 6 months were exclusively breastfed [11]. These improvements in MIYCN practices can be partly attributed to strategies such as the Baby Friendly Community Initiative (BFCI), which aims to promote MIYCN at the community level, delivered primarily by community health volunteers who provide counselling and support to pregnant and lactating women [12].

The BFCI builds on the baby-friendly hospital initiative (BFHI)’s 10 steps of policies and procedures required to support breastfeeding at the health facility level [13]. The impact of the BFHI was limited because a significant proportion of women still deliver at home, and even those who deliver in the hospital are discharged home quickly and require a continuity of care at the community level [11]. As a way to address this problem, Kenya adopted the baby-friendly community initiative (BFCI) - a World Health Organization-recommended strategy to promote optimal MIYCN at the community level [12]. The BFCI applies the principles of the BFHI by extending follow-up and care of the mother and child to the community. The strategy is currently being implemented across the country and is effective in promoting exclusive breastfeeding. It includes eight implementation steps which include: having a written MIYCN policy summary statement that is routinely communicated to health providers, community health volunteers and community, capacity strengthening of health care providers to implement the MIYCN policy, promotion of optimal maternal nutrition to women and their families, sensitizing mother and their families about the benefits of breastfeeding and the risks of artificial feeding, supporting mothers to initiate breastfeeding within the first hour of birth, establish and maintain exclusive breastfeeding for first 6 months, encourage continued breastfeeding beyond 6 months and timely introduction of appropriate complementary foods, provision of a conducive environment for breastfeeding families and promote collaboration between health staff, the local community and support groups [14].

The BFCI aims at providing health care workers and community health promoters with skills to support mothers to optimally feed their children. The package includes maternal nutrition and health counselling messages, importance of EBF and the key processes including early initiation to breastfeeding, feeding of colostrum and attachment and positioning. Mothers are also counselled on ways of preventing mother to child transmission (PMTCT) of HIV, solving breastfeeding difficulties and obtaining family support. In addition, the strategy includes community mother support groups which oversee, plan and execute community meetings on the BFCI; mobilize all community members to participate in BFCI activities; support and mother to mother support groups which provide peer support to breastfeeding mothers.

Given that the pandemic has led to limited access to child health services like growth monitoring, community services such as the BFCI may have also been affected but to date there is limited evidence presented in the literature of this in Kenya [5]. We undertook a qualitative study to gain a better understanding of the impact of COVID-19 on the BFCI and MIYCN in Kenya at multiple levels, county, sub-county, community and individual level (mother and child), which occurred during the pandemic. This study aims to assess the impact of COVID-19 on the functioning of the BFCI and other maternal and child health services in Kenya. It also aims to investigate how the pandemic impacted the nutrition and feeding practices of women and children in the country with the view to identifying gaps in the health services and to consider guidelines that can improve mother and child health outcomes in the face of similar pandemics in the future.

## Methodology

### Study settings

The research was conducted in three main counties in Kenya: Nairobi, Kiambu, and Baringo, which include both urban and rural BFCI implementation areas and non-BFCI areas. The BFCI implemented areas selected were Nairobi-Dagoreti (urban), while rural regions were Baringo-Koibatek and Kiambu-Lari. Ruaraka in Nairobi was selected as an urban non-BFCI implementing area while Kiambu-Gatundu was selected as a rural non-implementing area. In selecting the sub-counties, we considered logistical issues in conducting field work given the available resources and the prevailing COVID-19 situation. Koibatek sub-county was selected because of our previous work on BFCI in the sub-county. Rural and urban areas were considered because of the potential differences in challenges and experiences in implementing BFCI in the two settings. In 2014, childhood stunting rates in Baringo, Kiambu and Nairobi were 29.5%, 15.7% and 17.2%, respectively [11]. The median breastfeeding duration was 3.1 months in Rift Valley where Baringo is located and 4.3 months in Central where Kiambu is located [11]. Home births are also relatively common in Rift Valley where 49% of women deliver at home compared to Kiambu where 9% of women deliver at home [11].

### Study design, sample size and sampling strategy

This cross-sectional study collected qualitative data from October to November 2020 through 51 Key Informant Interviews (KIIs), 31 In-depth interviews (IDIs), and 15 Focus Group Discussions (FGDs) in each site. Each FGD was limited to a total of six members so that social distancing could be observed. A total of 103 interviews comprising 205 participants were conducted. A summary of the number of interviews conducted is presented in Table 1. The number of focus groups and interviews to be conducted was determined by the number of targeted stakeholders to ensure that we captured insights on BFCI implementation from different perspectives.

**Table 1:** Number and types of interviews conducted by study site

	BFCI Implementing			BFCI Non - implementing			
	Rural		Urban	Rural	Urban	National Level	Total
	Koibatek	Lari	Dagoretti	Gatundu	Ruaraka		
KII	10	10	11	8	8	10	57
IDI	8	6	5	6	6	-	31
FGD	3	3	3	3	3	-	15
Total	21	19	19	17	17	10	103

Purposive, snowball, and stratified sampling techniques were used to identify study sites and recruit eligible participants to this study. Purposive and stratified sampling was used to identify and select BFCI implementing and non-implementing sites in rural and urban areas to allow for comparison between the sites. Koibatek was specifically selected because of our previous work on the effectiveness of BFCI on exclusive breastfeeding [14]. Relevant stakeholders involved in the BFCI implementation activities at all implementation levels (national, county, sub-County, and community level) were identified using stratified sampling. Snowball and purposive sampling were used to select the most relevant stakeholders from each stakeholder group to participate in the KII and IDIs. Purposive sampling was used to select FGD participants who included caregivers (mothers, fathers, or primary caregivers) and community health volunteers. Community leaders and community health volunteers assisted in the identification of participants for the FGDs.

Participants included in the study were included if: they were part of the BFCI implementation process for settings where BFCI is implemented. In non- BFCI implementing areas, we worked with community health volunteers to identify caregivers of children under 5 years of age, people within the community who have knowledge on nutrition and food supply, people who could contribute to discussions around maternal and child nutrition practices in the community.

### Data collection procedures

KIIs were conducted with national, county, and sub-county stakeholders, and with relevant community leaders, health facility managers, and implementing partners from non-governmental organizations (NGOs). The interviews focused on understanding participants’ experiences and perceptions on the impact of COVID-19 on MIYCN and BFCI implementation activities and what could be done to improve the situation during the pandemic. Participants from counties that were not implementing the BFCI in Kenya were also included to understand experiences of influences of services targeted at MIYCN both in counties that were implementing and not implementing the BFCI as the BFCI had not rolled out nationally at this time.

The IDIs were mainly held with community members who were likely to influence MIYCN practices at the community level and those involved in the BFCI activities. The interviews were conducted to complement information obtained from the FGDs and KIIs on the impact of the COVID-19 pandemic on MIYCN practices and BFCI implementation activities, taking a high-level view of the practices at the national and sub-national level.

KIIs with different stakeholders were conducted virtually using phones and online meeting applications to minimize face to face contact whilst COVID-19 restrictions were still operating. IDIs with community members were conducted over the phone or face-to-face while FGDs were conducted face-to-face. In all face-to-face interviews, COVID-19 prevention guidelines were observed.

### Data collection tools and data management

Interview guides were designed to ensure that relevant questions were asked. The questions asked covered the effects of COVID-19 on: the BFCI, child feeding and care as well as potential actions that can reduce the impact of COVID-19. The qualitative tools were pre-tested by field interviewers to evaluate the validity of the interview guides. The interview guides were amended based on the feedback from the pre-test.

Key informant interviews and indepth interviews were conducted in English while focus group discussions were conducted in Swahili. The data collection tools were back translated to ensure uniformity in the terminologies that were used.

Data quality control measures included conducting regular debriefing sessions between the investigators and field interviewers to ensure that emerging ideas were followed up in subsequent interviews. Transcripts also underwent data verification which ensured accuracy against the original audio recordings.

### Ethical considerations

The study was conducted in compliance with international and local ethics guidelines. Principles guiding research on human participants including respect for persons, justice, beneficence, and non-maleficence were observed. Ethical approval was sought from the AMREF Ethics and Scientific Review Committee (P843-2020). Informed written consent was obtained from study participants.

### Data analysis and presentation

Recorded audio files were transcribed verbatim, anonymized, stored in rich text format and imported into NVivo 12 software (QSR International Pty Ltd, Don Caster, Victoria, Australia) for coding and thematic analysis. A codebook was developed and used to guide the coding. Key content areas and codes in the codebook were determined deductively based on anticipated barriers and facilitators in the BFCI implementation [15]. Additional codes that came up during the analysis were also included. A quality assessment of the coded data was conducted by the research team. Final checks for consistency of the application of the codes was undertaken by a member of the research team.

## Results

Several themes related to the impact of the COVID-19 pandemic on the BFCI and other MIYCN emerged from our analysis. Our findings are presented under three separate headings related to the main themes: service disruptions due to COVID-19, impact of COVID-19 on BFCI and impact of COVID-19 on MIYCN.

### Service disruptions due to COVID-19

The impact of the pandemic on the provision and accessibility of maternal and child health services varied depending on the nature of the service as well as the service provision location: whether they were facility-based or home/community-based.

Disruptions to service provision were most common among MCH services that were strictly provided by health care facilities and required mothers and children to be present physically, such as facility-based vaccinations and scheduled clinical check-ups. This was because of health facility closures at the peak of the pandemic. Health facilities that were not closed had to pare down service provision due to reduced staff numbers from fears of COVID-19 infection, and inadequate infrastructure to ensure that health facility spaces were COVID-19 safe, including lack of personal protective equipment (PPE) for staff, and limited physical space to ensure physical distancing. Clinic attendance by mothers was also low due to fears of contracting COVID-19 and admission into quarantine if their children showed signs of fever (Table 2).

**Table 2:** Summary of quotes highlighting disruption of facility and community health and nutrition services

Service Disruptions during COVID-19	Supporting Quotes
Disruption of MCH services	*“Let’s say in the facility we normally provided health education to mothers*,* when COVID-19 was announced the number reduced [coming to the facility] almost to zero for the first few days. Most of the children were not coming for growth monitoring. They (mothers) feared*,* if the mother has fever*,* they will be admitted and taken for quarantine*,* so they feared a lot*,* so attendance was poor.****KII***,*** nutritionist***,*** Gatundu***,*** BFCI non-implementing rural site***
Reduced clinic attendance due to fear of contracting the virus	*“When COVID came*,* most of the parents could not visit the facility. If for example*,* you are talking about the child welfare clinic*,* the parents*,* could not continue attending because people believe that now the facility or the hospitals were institutions carrying the COVID…they could not continue with their immunization monitoring growth and the rest. Even those on some kinds of therapies could not continue.”****KII***,*** community health representative***,*** Lari***,*** BFCI implementing rural site*** *“Some of the clients feared that if they come to the dispensary*,* they may contract COVID-19*,* so some of them took time to come to the dispensary for even the growth monitoring.”****KII***,*** health worker***,*** Koibatek***,*** BFCI implementing rural site***
Disruption of community health services	*“There is what we call “Malezi Bora”. We do it in May and September. Basically we do it in schools and homes. Around May when cases were high*,* we were not able to reach as many children as we would like to because many people feared interactions with people who they don’t know.”****KII***,*** community health assistant***,*** BFCI non-implementing rural site***

MCH services that involved home visits fared better than hospital-based care but were not altogether spared from pandemic-related disruptions. Home visitations were also disrupted by such factors as lack of PPE for CHVs, mothers’ fear of contracting COVID-19 from CHVs and CHVs’ fear of contracting COVID-19 from households. The “Malezi Bora”, an initiative that aims to accelerate the utilization of maternal and child health and nutrition services offered in county health facilities, registered a decline in the number of children that were reached during the pandemic (Table 2).

### Impact of COVID-19 on the functioning of the BFCI

The pandemic negatively impacted the functioning of BFCI activities. It affected monthly meetings, targeted CHV visits, dialogue days, support supervision and support group meetings by either reducing their frequency and attendance numbers or pausing the activities completely. Evidence from our data suggests that the pandemic affected certain programme components of the BFCI, such as registration of mothers into the programme, data collection and reporting. The main reasons affecting the functioning of the BFCI can be attributed to three factors: participants’ fears of contracting COVID-19, non-availability of PPEs and movement restrictions (Table 3). As a result, CHVs could not access households, while some mothers in urban areas moved to their rural homes and were therefore not accessible.

**Table 3:** A summary of factors which led to the disruption of BFCI activities

Causes of disruptions on BFCI Activities	Supporting quotes
Fear of contracting COVID-19	*“We have been called here to bring children*,* there is fear because I don’t know if the one I am going to meet is sick or not then if they call for a meeting here it is a problem because someone has that fear*,* you see maybe I am going to take my child there and get Corona. So*,* people have fear*,* we have had a lot of fear*,* where people are crowded when you hear they are calling for a meeting you get scared*,* should I go with the child and if the child gets Corona*,* what will I do”****IDI mother***,*** BFCI implementing urban site*** *“In fact*,* in March there was little work being done by CHVs*,* because*,* of that fear*,* people did not want visitors in their compound”****KII Community health strategy representative***,*** BFCI implementing rural site***
Movement Restrictions	*“… In fact*,* some (mothers) disappear*,* they just come once and they disappear completely. So*,* tracing them*,* like I told you*,* you call and find that the number is not in service*,* or you are told it’s a wrong number. So*,* some go…maybe they come after a month or two months and when you ask them*,* they will tell you they went back to their rural homes. And that time of lockdown*,* some of them were locked. So*,* when they came*,* their children had deteriorated.”****KII with a nutritionist***,*** BFCI non-implementing urban site*** *"“… the moment COVID came*,* people went to different places*,* some returned to their rural homes when they lost money immediately movement restrictions were opened*,* many people went back to the village. Right now*,* we are trying to regroup and see if they may be found and we are trying to recruit new membership.”****IDI with a lactating mother***,*** BFCI implementing urban site.***
Lack of PPEs	*“Things are tough because me as a CHV I cannot visit that house since we were not supplied with personal protective equipment’s*,* so it became a challenge”****FGD***,*** community health volunteers***,*** BFCI non-implementing rural site*** *“Home visits were also affected because we wouldn’t give the CHVs protective gears*,* maybe if they were given by some organization then it would work but most of them were not provided with protective gears.”****KII with a non-governmental organization representative***

### Impact of COVID-19 on maternal & child nutrition and feeding practices

The pandemic also negatively affected maternal and child nutrition and feeding practices mainly through its impact on the household finances and livelihoods. Job losses that occurred as a result of the pandemic led to a decrease in incomes and left many households unable to purchase nutritious food. As a consequence, coping strategies such as skipping meals were reported by our study participants (Table 4). In addition, due to financial difficulties, some mothers were unable to practice appropriate complementary feeding.

**Table 4:** Summary of the impact of COVID-19 on maternal and child nutrition and feeding practices

Impact of COVID-19 on nutrition and feeding practices	Supporting Quotes
Impact on livelihhods, food insecurity and coping strategies	*“Because they could not maybe afford to purchase food that is nutritious for children who are 6 months and above and are also themselves mothers*,* who are pregnant or lactating*,* they were not able to feed themselves*,* instead of taking the three or five meals per day they were taking two meals per day*,* so*,* they were skipping some meals. Or also some were giving maybe more rations to other children or giving more attention to other children than the others”****KII***,*** nutrition officer***,*** BFCI non-implementing urban site*** *“The mother has no food to generate breastmilk. Or those who are now on complementary feeds*,* there’s no food to eat. What do you eat? So*,* the baby has not been thriving properly. They are going back to malnutrition. COVID-19 led to job loss; most people were terminated. Like tea pickers in this area lost their jobs because that job is now done by the family e.g.*,* like students who are at home. Now you find that person who lost her job will not achieve good nutrition for the child because she cannot afford to buy things like fruits or other luxury commodities due to lack of money.”****KII***,*** food vendor***,*** BFCI non-implementing rural site***
Impact on breastfeeding	*“But still others benefited*,* because for example teachers and workers who were working at home. They were able to breastfeed up to six months*,* yeah*,* unlike when they are working you know some lose their babies at three months*,* so exclusive breastfeeding did well.”****KII***,*** nutrition officer***,*** BFCI implementing rural site***

Factors related to mothers’ mental health and wellbeing also affected negatively maternal and child nutrition and feeding practices. For example, some women opted not to breastfeed their children, especially after work because of stress, inadequate food, and fear of infecting infants with COVID-19. Mother’s employment arrangements impacted their feeding practices. Women in formal employment had the opportunity to breastfeed their children, impacting positively on breastfeeding. This was attributed to COVID-19 containment measures such as working from home, curfew hours and lockdown, which meant that they spent more time at home with their children and this improved breastfeeding practices (Table 4).

## Discussion and recommendations

This study aimed to assess the impact of the COVID-19 pandemic on the BFCI, maternal and child health services and infant and young child feeding practices. We found that access to and provision of MCH services such as antenatal and child immunization decreased during the initial stages of the pandemic. Some BFCI activities such as CHV home visits and community meetings were also negatively affected. The disruption of these activities was attributed to fear of contracting COVID-19 by both mothers and health staff, low health staff numbers due to lack of PPEs and movement restrictions. Similar findings have been reported by other studies [2, 5, 16–18]. In a study assessing the impact of the imposed lockdowns and curfew on access to maternal health services for women living in informal settlements, women reported limited access to health facilities due to fear of contracting the virus from health staff and other people in the clinic [5]. Kotlar, Gerson [18] noted that maternal and perinatal programs in low-, middle-, and high-income countries were faced with similar problems during the pandemic. However, while many high-income countries were able to adapt their programs to continue to offer services during the pandemic, programs in many LMICs had to scale down service provision and our study findings support this [19].

The pandemic had both positive and negative effects on breastfeeding. In some cases, women had limited access to food and limited time for child care which resulted in poor breastfeeding practices while others, because of the COVID-19 restrictions, had more time at home and were, able to feed their children. These findings highlight the need for proper support structures for breastfeeding women during pandemic conditions. These findings were in line with recent international publications [2, 9, 20, 21]. The fact that women working from home had an opportunity to breastfeed and bond with their infants shows the potential for implementation of flexible working hours as well as hybrid working arrangements that allow women to work both remotely or in hybrid working patterns as a strategy for supporting breastfeeding in the workplace. Such strategies warrant the revision of documents such as the Kenya’s Health Act in preparation for post-pandemic life [22].

Food insecurity was also a challenge due to the loss of income sources as a result of the pandemic, which in turn affected access to nutritious foods by pregnant women, mothers and children. Coping strategies such as skipping meals were reported. Similar findings have been reported by other studies conducted during the COVID-19 pandemic in Kenya and Peru [2, 3, 5, 23, 24]. This shows the need for social protection of vulnerable groups, especially women and children. Although the government of Kenya provided social safety nets in the form of cash transfers, this was a short term measure and not all vulnerable groups had access to this type of support [25]. Other social support systems may have been put in place in the study areas by other institutions, but these were not documented. There is also a need to improve food systems not only in Kenya but also globally to make them more sustainable and equitable given that food insecurity experienced during this period was mainly systemic.

Gaps in the health system were also highlighted by the lack of PPE for health staff and CHVs. To ensure continuity of care during a pandemic, proper structures need to be put in place to ensure that health care providers are protected including the provision of PPE.

Although we were able to document the impact of the COVID-19 pandemic on BFCI, MCH and MIYCN, we did not document all adaptations that were made in the delivery of community health and nutrition services.

## Conclusions

The findings from this study highlight the negative impact of the COVID-19 pandemic on the BFCI, MCH services and infant and young child feeding primarily due to fear of contracting the virus, movement restrictions and lack of PPE. Moving forward, proper information structures and infrastructure should be provided to ensure continued provision of and access to healthcare regardless of the prevailing infection risk situation. Work policies should be reviewed to consider further evidence for the role of remote working policies to improve breastfeeding among working women.

## Data Availability

The data that support the findings of this study are available upon request from the African Population and Health Research Center, Microdata portal. Please reach out Antonina Mutoro- amutoro@aphrc.org for more information.

## Abbreviations

AMREF: African Medical Research Foundation
BFCI: Baby friendly community initiative
CHVs: Community Health Volunteers
LMIC: Low and Middle Income Countries
IDI: Indepth Interview
KII: Key informant interview
FGD: Focus Group Discussion
MIYCN: Maternal Infant and Young Child Nutrition
MCH: Maternal and Child Health
PPE: Personal Protective Equipment
